# Fallacies of the Faculty; with the Principles of the Chrono-Thermal System. In a Series of Lectures

**Published:** 1843-01

**Authors:** 


ART. X.

1. *Fallacies of the Faculty; with the Principles of the Chrono-thermal System. In a Series of Lectures.* By SAMUEL DICKSON, M.D., late a Medical Officer on the Staff. Second Edition.—*London,* 1841. 8vo, pp. 328.
2. *Erreurs des Médecins, ou Systême Chrono-thermal; traduit de l'Anglais du Dr. Dickson.* Par MALVIUS, A.D.C.—*Paris,* 1842. 8vo, pp. 560.

THE author of this publication insists that the medical profession, as a body, are "swindlers," and something worse; that "the whole *régime* of medical teaching is a system of humbug, collusion, and tricks;" and "that the notions which have hitherto guided physicians in their treatment of disease are a mere romance." He proposes to work a thorough revolution in medical science and morals; and, while he exposes the fallacies and crimes of the faculty, humanely sets forth an absolutely novel, correct, and complete system of medicine of his own. He is evidently a person of infinite magnanimity. He feels convinced that he will be a martyr to his faith; but, fearlessly classing himself with those persecuted lovers of truth, the Harveys, Newtons, Parès, Jenners, and Lady Mary Montagues, he awaits his fate with the heroism becoming his great mission. Being ourselves persecuted lovers of truth, we shall examine the new system of Dr. Dickson in a dispassionate spirit, and notice the other little points respecting medical ethics as we have space.

Our author (in the name of truth) appeals to "nature, eternal nature. By this," he remarks, "and this only, am I willing that the new fabric of medicine which I have presumed to erect upon the ruins and reveries of the past, should be tested and tried;" pathetically adding "till the world shall detect one real, one indubitable fact militating against the views I am now about to develop, let not innovation be charged against me as a crime." This preliminary squeak for mercy comes in rather awkwardly, to say the least of it. The world will most likely look upon Dr. Dickson's innovation as a pleasantry; and certainly, we shall seek for no militant facts, but simply confine ourselves to ascertaining whether the facts adduced in its support by Dr. Dickson himself are of the right sort and convincing. Dr. Dickson cannot desire us to do less.

This new system is termed *chrono-thermal,* because it refers to time and temperature. There can be no increase or diminution of temperature without motion, no motion without time; motion consists in attraction and repulsion; attraction and repulsion are peculiar to electric action; *argal,* medicines must change the motions of the system, and must be electrical in their operation. But our author shall state his own views. Having laid down the phenomena of health, he observes:

"If we analyse these various phenomena, we shall find that they all consist in a series of alternate motions, motions for the fulfilment of which, various spaces of time are requisite; some being diurnal, some recurring in a greater or less number of hours, while others exhibit a minutary or momentary succession. At morn, man rises to his labour; at night he returns to the repose of sleep; again he wakes and labours—again, at the appointed period, he 'steeps his senses in forgetfulness' once more. His lungs now inspire air, now expel it—his hear successively contracts and dilates—his blood brightens into crimson in the arterial circle of its vessels—again to darken and assume the hue of Modena. The female partner of his lot, she, who shares with him the succession of petty joys and sorrows, hopes and fears, which make up the day-dream of life, has yet another revolution, the *catamenial;* and *parturition*, or the process by which she brings their mutual offspring into the world, is a series of pains and remissions." (p. 11.)

Every atom of the material body is constantly undergoing a revolution or alternation at regular periods. "It is everything by turns, and nothing long," remarks Dr. Dickson.

"Gentlemen, There can be no motion in matter *without change of temperature*, and no change of temperature *without motion in matter.* This is so indisputable an axiom in physics, that Bacon and others supposed motion and change of temperature to be one and the same.......If even in the middle of winter, you run for any length of time, you shall become heated and bloated; and you again shrink in size when you stand still to cool yourselves. Those who ascribe the source of animal heat exclusively to the lungs, seem to have forgotten these facts: they have forgotten, that in the constant mutation of its atoms, every organ, nay every atom of that organ, being ever in motion, must equally contribute to this end; for to this common law of ALL matter, every change in the body is subjected.* The powers by which the corporeal motions are influenced, are the same that influence the motions of every kind of matter, namely, the electric, mechanical, and chemical forces, and the force of gravitation. When rightly considered, the whole of these powers resolve themselves into ATTRACTION and REPULSION. It is by *attraction* that the fluid matter of the blood first assumes the solid consistence of an organ; again to pass by *repulsion* into the fluidity of secretion." (p. 12.)

This is an exposition of the first principles and physiology of the *chrono-thermal* system. The pathology is thus expounded: "Whatever be the cause or causes of corporeal aberration, in obedience to the law of all matter, the first effect is a *change of temperature.* The patient accordingly has a feeling of heat or cold." But our author must state his own views:

"Gentlemen, We have already analysed the Life of Health. If, in the language of Shakspeare, it be indeed a '*fitful* fever,' what can the morbid modifications of that life be, but modifications of FITFUL OR INTERMITTENT FEVER? ............ If we succeed in proving to you that asthma, epilepsy, toothach, gout, mania, and apoplexy come on in *fits;* that all have febrile *chills* or *heats;* that remissions or periods of immunity from suffering are common to each; and that every one of them moreover *may* be cured by any one of the agents most generally successful in the treatment of INTERMITTENT FEVER, popularly termed AGUE; to what other conclusion can we possibly come, but that this same AGUE is the type which pervades and the bond which associates together every one of these so-called different diseases? But if in the course of these lectures we further prove that what are called 'inflammations' also come on in fits; that they have equally their periods of immunity from pain, and yield with equal readiness to the same remedial means, who can be so unreasonable as to doubt or dispute that AGUE IS THE TYPE OF ALL DISEASE?" (p. 17.)

Aye, Who? But then there are those IFS; and as a sceptical reader may exclaim, "in the language of Shakspeare,"

"Talk'st thou to me of *ifs?*"

Dr. Dickson furnishes proofs sufficient—in number, at least—for the

* In this and all subsequent quotations we give the *capitals* and *italics* of the author just as we find them in the book.

most sceptical. This, the first lecture, closes with a promise to prove in the next that "asthma, epilepsy, palsy, curved spine, squint," &c. are merely so many developments occurring in its [an ague's] course; *analytically*, by rigidly scrutinising their symptoms; *synthetically*, by detailing cases of each cured on CHRONO-THERMAL principles. We shall note the cases and pass over the analytical argumentation. We would observe, however, that the author commences this latter by assuming that he has "proved, that healthy life is a FITFUL FEVER," on the authority of Doctor Shakspeare, we suppose, as there is no other proof of this great fact than what is contained in the passages placed before our readers. It is true that we have a passage in which the human system is compared to our globe; and its vital mutations, "such as tumours, abscesses, and eruptions," to the "new-formed mountain-masses, earthquakes, and volcanos" of our mother-earth. But to make this argument valid, it should have been proved that all the earth's changes are developments of an ague. This Dr. Dickson does not prove. His great authority, Dr. Shakspeare, appears, indeed, to hint that the earth is liable to attacks of intermittents, at least to the *chilly* or shaking stage. Glendower says:

"At my birth,
The frame and huge foundation of the earth
*Shaked* like a coward."

According to the same writer the earth suffers from "windy colic" sometimes; which disease, chrono-thermally speaking, is an ague. The following are cases in "proof" of the *thermal* portion of Dr. Dickson's system:

"Sir R—— A——, while serving in Portugal, became the subject of severe ague, which resisted a host of remedies prescribed for him by numerous medical friends—bark among the number. One day when riding out he was seized with a paroxysm. The inmate of a little shop where he dismounted till the fit should be over, suggested to him to try the barber-surgeon of his neighbourhood. .... ........ The ambidexter man of medicine came, ordered him a large plaster to his back, and the ague was forthwith cured! Gentlemen, to what but to the improvement of the *temperature* of the spine must we attribute the success of the plaster?" (p. 27.)

"I have said that ASTHMA is a remittent disease. In one case, that of a gentleman who had the disease every second night: after having nearly exhausted all my best resources, I succeeded in curing him by the application of a *warm* plaster along his spine. Here you again see, in the most direct manner, the advantage of attention to *temperature*. The spine in this case was always chilly, but became warm and comfortable under the use of the plaster." (p. 30.)

The great majority of urethral strictures yield readily to "*chrono-thermal*" treatment in Dr. Dickson's hands. Bougies, those cruel instruments, used by none but dishonest persons, may be almost dispensed with. Curved spine, which Stromeyer and a few other insignificant schoolmen, have attributed to paralysis of certain sets of muscles, is also in the opinion of Dr. Dickson a remittent affection; proved as follows:

"In the commencement of most cases of this kind, the patient is taller one day than another, a proof of remission; and I have never had such a patient who has not confessed to heats and chills. I will give you two cases in which these phenomena were observed:

"A young lady, aged sixteen, had a lateral curvature of the dorsal vertebræ, causing the inferior angle of the shoulder-blade to protrude. I prescribed for her calomel and quinine, in small doses, and directed her to have her spine rubbed night and morning with soap liniment. In less than a month, the patient gained three inches in height, and in two months more she was erect." (p. 37.)

The other case was that of a lady, aged forty-five, who "stood upright" in three weeks. Squint and amaurosis are proved to be merely developments of remittent fever, and yield readily, of course, to chrono-thermal remedies, pregnancy being one of these. Deafness is in the same category, and so are bulimia, &c.

"If patients who are subject to DEAFNESS be asked whether they hear better upon some days than others, the great majority will reply in the affirmative. So that deafness also is a remittent disease. That it is moreover a feature or development of general constitutional disorder is equally certain, from the *chills* and *heats* to which the great body of patients affected with it, acknowledge they are subject." (p. 41.)

"A gentleman, who was fond of play, told me that when he lost much money he was always sure to become *ravenously hungry;* but that when he won this did not happen. The *temperature* of his body must have been different at different times." (p. 42.)

If we were to go through the cases of hemorrhage, apoplexy, heart-diseases, gout, stone, consumption, &c. we should only multiply cases resembling the preceding. Dr. Dickson is satisfied no one ever explained the real nature of phthisis before himself. The relation of that disease to ague is clearly established by "*chills and heats,*" and the similarity of modes in which the two diseases may be cured. Dr. Dickson observes, "with quinine and other chrono-thermal medicines, I am satisfied I have cured or arrested at least 500 cases of consumption, many of them, too, in apparently advanced stages." Scrofulous joints are cured, we may say, "in a winking."

"Harriet Buckle, seven months old, had what is called a scrofulous elbow. The joint was much enlarged, red, painful, and pervious to the probe, with discharge. The patient was the subject of *diurnal fever.* . . . . . . . . A powder, containing calomel, quinine, and rhubarb, in minute doses, was directed to be taken every third hour. The case was completely cured in a fortnight without any external application." (p. 79.)

Another case is a girl, aged twelve, was cured in six weeks. All kinds of inflammations are proved in this manner to be agues, and readily curable by chrono-thermal medicine, whether the so-called inflammation be in the brain, or bowels, or lungs, or heart, &c. Bloodletting of every kind is not only denounced as unnecessary and cruel, but convincingly shown to be impious; thus:

"Has not nature done everything to preserve to animals of every kind

'*The electric blood* with which their arteries run!'—BYRON.

She has provided it with strong resilient vessels,—vessels which slip from the touch, and never permit their contents to escape, except where their coats have been injured by accident or disease. Misguided by theory, man, presumptuous man, has dared to divide what God, as a part of creation, united—to open what the Eternal, in the wisdom of his omniscience, made entire! See! what an *extreme* this is! It is on the very face of it a most unnatural proceeding," &c. (p. 99.)

The profession ought to have a general fast, and all day long repeat the litany, as one method of escaping the vengeance due to their impious presumption in phlebotomising their patients. Abstinence is also noticed as a dangerous thing. "Beware of carrying this too far!" says our author, "for abstinence engenders maladies: so Shakspeare said, and so nature will tell you, in the teeth of all the doctors in Europe!"

Smallpox is one of the few diseases which our author does not perfectly comprehend. He queries whether vaccine lymph be, "as Linnæus thought, of an animalculine character? or is it at all analogous to the influence produced by the magnet on iron?" Dr. Dickson has been in the profession for seventeen years, and declares he never saw syphilis, although eight of those years were spent in the army. There is no such thing; the series of phenomena ignorantly termed syphilis, being simply developments of ague, and curable by chrono-thermals, as iodine, calomel, &c.

Mania only differs in degree from a violent fever. Both have remissions, both are cured by the same remedy, namely, a ligature to a limb. "Mania, epilepsy, asthma, cramp, AGUE, then," writes Dr. Dickson, "completely establish their fraternal relationship by means of the ligature." Is not the relationship of ague (we would ask Dr. Dickson), rather paternal or *maternal*, than fraternal? or is it *conjugal?*

Chlorosis, menstruation, and leucorrhœa are all modifications of intermittent fever; and the diseases are to be cured by chrono-thermal remedies; a *warm* plaster, for example, to the spine in leucorrhœa. Cancer is in the same category; it is an ague.

"Analyse this disorder [cancer], and you will find it resolve itself into a general intermittent febrile action of the whole body, varying in its shade with every case. Cancer, then, is a development of that fever. Gentlemen, never in my life did I meet with a cancer in any state or stage, the subject of which did not acknowledge to chills and heats, or who did not admit errors of secretion." (p. 214.)

All tumours are agues:

"In the course of my professional career, I have witnessed tumours of every description, but I never met one that could not be traced to previous constitutional disturbance. ............ Chills, and heats have been confessed to by almost every patient, and the great majority have remembered that in the earlier stages, their tumour was *alternately* more or less voluminous." (p. 219.)

Pregnancy and parturition are agues:

"Pregnancy *is* a natural process; but is it on that account the less surely a *febrile* state? Is it for that reason the less certainly an *intermittent* fever?" (p. 223.)

Pregnancy will cure a squint; Dr. Dickson witnessed a splendid "case;" and therefore this state harmonizes completely with the unity which pervades disease generally. Parturition, too, is a series of pains and remissions:

"In spite of every assertion to the contrary, then, in spite of every declaration on the part of medical or other persons, pregnancy and parturition *are* agues—agues in every sense of the word; for not only do their revolutions take place in the same identical manner as ague, but like agues, they may be influenced by medicines as well as by mental impressions." (p. 224.)

The passions are agues. Love is most certainly, because Hudibras said it was an ague-fit reversed; the hot stage coming first. The earth in a fright is in a fever, has a regular ague fit. Medical practitioners who go and see their patients with long lugubrious faces, do the trick on set purpose. They know well that fear is an ague-fit, and therefore retards convalescence, much to their pecuniary benefit. "The mode of its action establishes beyond cavil," Dr. Dickson observes, "not only the unity of disease, but the unity of action, of remedy, and cause." Baths and plasters are useful because they are *warm;* bandages because they alter the motions and temperatures of parts; ointments because they are but combinations of the agents with which we combat fever.

Hahnemann and homœopathy are unmercifully scouted by Dr. Dickson; a fact exhibiting a proof of the wisdom of Shakspeare, when he wrote

"Tut man! one fire puts out another's burning."

Ague and Hahnemann being here in opposition, and extinguishing each other. Dr. Shakspeare, our author insists, discovered homœopathy, and not the impudent Hahnemann.

We have not room for the proofs Dr. Dickson adduces, that the action of *medicinal* agents is purely electrical; but they resemble all his other proofs. The doctrine is extended to animal tissues.

"You may with the most perfect propriety ask, why the influence of opium on the brain should set one man to sleep and keep another from sleeping?.... ........ The answer is simple, and affords a fresh illustration of the electrical doctrine. The atoms of the specific portion of the brain of any two individuals thus oppositely influenced in either case, must be in *electrically opposite* conditions, *negative* in one, and *positive* in the other." (p. 284.)

Dr. Dickson adds, "the merit of this explanation I exclusively claim. —Yes! Gentlemen! I exclusively claim the ELECTRICAL DOCTRINE of medicinal agency as mine."

To us the electrical doctrine is stark staring nonsense; but this may be because we are schoolmen and, as far as our ability would permit, have studied electricity and organic chemistry. This doctrine, however, explains the *modus operandi* of all medicinal agents. For example:

"IODINE. Gentlemen, like every other remedial agent it cuts two ways, anatomically *attracting* or lessening volume and secretion in one case, anatomically *repelling* or increasing both in another, according to the *electric* state of the body for which it may be prescribed." (p. 294.)

"ALUM. The earth called alum is a favorite with the common people in the cure of ague. What is its mode of action? Its power of astringency or attraction simply—the same power by which it arrests the morbid increase of secretion, called leucorrhœa. How does it do that? By its attractive influence over the atoms of the spine, and the nerves proceeding from the spine. Well, then, that is the way in which it cures ague." (p. 299.)

We can only exclaim, lost in admiration and wonder, "What it is to be a scholar!"

The lapses and backslidings to which professors of religion are subject are influenced by electricity, and are ("as one may say,") *agueish.* Shakspeare proves it.

"That excess of religious feeling, or *veneration* (as the phrenologists call it), does, however, depend upon the temperature or motive condition of some cerebral part, there cannot be a doubt; and that it takes place by fits or periods, Shakspeare well knew, for he makes one of Clarence's murderers say,—'I hope this holy humour of mine will change; it was wont to hold but *while* we could count twenty.'" (p. 312.)

So that Shakspeare not only anticipated Dr. Dickson in periodicity, but has given the measure of the periods; namely, "while one could count twenty;"—a great deal more than our modest author has ventured on.

Our readers will, by this time, have formed an opinion of Dr. Dickson's publication. We have done justice to his doctrines, by giving them and the proofs in his own language. The plain truth is, as every one must see, the whole book is a farrago of nonsense; a hash of a few old truths and many fantastic speculations, made piquant by the most amusing self-laudation on the part of its author, and the most extravagant abuse of his professional brethren and imagined rivals. It is clear that Dr. Dickson cannot have derived his knowledge of the foolish, murderous, and dishonest practices he attributes to ten or twelve thousand individuals, from personal knowledge; it can only have been obtained by his acquaintance with a few,—and bad ones they have been. So intimate is his knowledge of professional turpitude, that one cannot help thinking of the pithy adage, "Set a thief to catch a thief,"—but, of course, not thinking of applying it to him. Like "one of the wicked," as Falstaff says, who has become honest and turned informer, Dr. Dickson declares he will make people's ears tingle by his revelations, if provoked to desperate deeds. We hope our professional brethren will endure the exposure with fortitude. M. Malvius (who seems to have judged that the piquancy we have alluded to would suit the French market) observes, that Dr. Dickson's "clientel" is very numerous. We have made an approximative calculation on this point, on the plan of the new numerical method. We have reduced the various orientalisms in his publication, such as "many thousands," "hundreds," "a host," "dozens," &c., into plain numerals; and we estimate the total number of cases Dr. Dickson has treated at one hundred thousand. There are, to be sure, discrepancies. Dr. Dickson himself asserts, that, "out of upwards of TWELVE THOUSAND cases of disease that have, within the last few years, been under my treatment," *he has not been compelled to abstract blood* ONCE*;* but Dr. Fosbrooke, in a letter to Dr. Dickson, says, that in April, 1835, he pricked up his ears at Dr. Dickson's "repeated asseverations that in a practice embracing the treatment of *several thousands* of patients *per annum*, he never employed a lancet or a leech." We do not feel surprised that Dr. Dickson should state one year's practice only, as the practice of "the last few years." He is a modest man; and besides, it was necessary to be careful, lest he should be identified with the other "medical dissenters" who deal in thousands; as, for example, the authors of such an announcement as the following:—"Extraordinary sale of Parr's Life Pills; ONE HUNDRED AND SEVENTY-FIVE THOUSAND in one week!" To guard himself still more effectually against any imputation of this kind, Dr. Dickson very pithily and justly remarks, "Impostors *never fail!*" The diseases our author has really failed to cure or alleviate by bark, are only tetanus and hydrophobia. (p. 303.) Having failed in those he is, of course, no "impostor."

We freely accord Dr. Dickson the triple crown in modern science, as the greatest physician, philosopher, and poet of the age;—the Homer, Plato, and Hippocrates of modern times. Our tiara must, however, be made of *foolscap*, being the material nearest at hand, and of which we always make use when crowning great men like our author. Perhaps Dr. Dickson, being a man of warm feelings, may think our strictures severe, and, in the next edition of his book, may be inclined to be severe upon us. We crave his mercy, even for his own sake. We would advise Dr. Dickson to be mild, and eschew all symptoms indicative of a "fitful fever," lest—to quote the words of a little *chanson* in our memory (*not* one of Beranger's)—

"Lest people should cry
As he passes by,
There goes *Ague-Dick* in a flutter!"

If that expressive sobriquet should attach to our author, he might aptly exclaim, with *Ague*-cheek, in the "Twelfth Night,"—

"'Slight, do you mean to make an ass o' me?"

Certainly not, we would answer. But has not Dr. Dickson made an ass of himself? Aye, there's the rub!—"in the language of Shakspeare."

---

